# American Balneology

**Published:** 1917-05

**Authors:** Felix Von Oefele

**Affiliations:** New York


### American Balneology.

Dr. FELIX VON OEFELE, New York.

### THE CENTER OF UNITED STATES

The center of the United States contains few old fields of low mineralised springs, for instance, in the district of the Ozarks and Wisconsin. These fields correspond to islands emerged during the oldest eras and exhausted of soluble minerals. In general, the spring waters of this center are remarkably higher mineralised than the springs of the Atlantic areas.

The special eastern corners where two Atlantic areas border on the fourth area, are characterized by the two fields of thermal springs of the Tahonic Mountains and Virginia. These two thermal points indicate the straight direction of the eastern boundary line of the fourth area. There is a less practical reason to subdivide the Eastern Mississippi area into subareas according to the subdivided Atlantic areas.

The center of the United States forms the fourth, fifth and sixth balneological areas. In the time of Columbus they were wood land in their eastern part. The western Mississippi area overleaps to the prairies. The western Mississippi area shows prevailing homogeneous winds like monsoon; for the winter it is northwest and north, and for the summer southeast winds. The most annual rain falls near the Gulf of Mexico with kaenozoic and mesozoic groups of strata and properties for less valuable mineral springs. This amount of rain decreases slowly in the direction to the Ohio River in the eastern Mississippi area, but rapidly to the north in the western Mississippi area. When the amount of annual rain is decreased to a certain point, the condition of moisture for trees is gone, and the woodland changes to the character of woodless plains. The country of frequent rains and of woodland with palaezoic declines is rich in springs and the woodless plains are poor. This country rich in springs forms the fourth area of Eastern Mississippi districts.

The country west of the eastern American mountain system was at different times an enclosed gulf sea. The deposits are water deposits formed partly by concentration or evaporation. They contain a high amount of soluble constituents. At many locations the eastern Mississippi area contains sodium chloride deposits from an old palaeozoic sea, and at other locations calcium sulfate from later palaezoic periods. The southern part of these areas has kaenozoic origin. It will stay united in the fifth area. The western Mississippi area contains residues of some old palaezoic mountains; but the principal part is mesozoic deposit, rich in lime. Farther west, the Mountain area includes many rich salt kaenozoic concentrates of continental origin and young volcanic intrusions. The summation of these circumstances is the reason that the mineral waters of these three areas are commonly more highly mineralised than an average drinking water. The district of Wisconsin and the other Ozarks are exceptions of very old emersions with low mineralised springs.

### THE EASTERN MISSISSIPPI AREA.

The presence of a remarkable amount of sulfates and a condition of reduction within the higher strata very frequently create sulphide springs west of the eastern mountain chains. The inverted conditions are the reason for the absence of real sulphide springs in the three Atlantic areas. On this basis the metropolitan sick people of New York find the nearest sulfide springs, sodium chloride springs and calcium sulfate springs in the upper state between Albany and Buffalo. They depend upon these mineral springs, if they do not prefer a trip to the farther west. Only in western direction the patient from New York will find more mineral springs of high mineralisation. The same conditions are repeated for the inhabitants of Philadelphia, Washington and all cities of the thirteen oldest states.

AREA IV. District 1.

### CHAMPLAIN DISTRICT.

A small Cambrian and Ordovician ribbon passing through a small western part of New Foundland, is continued on the south shore of St. Lawrence River and forms the northwest corner of New England.

A connecting field from the submineralised springs of the New England area to the sulphide springs of the State of New York are the claimed mineral springs situated somewhat east of the Vermont sulphur springs. This is the field of the Cambrian rocks with calcium sulfate, especially at Chelton, Franklin County, Vermont, and vicinity, in which different springs of a certain type are located in about one mile radius.

By this type I mean low mineralised springs of more than 0.03% total solids, but with predominant calcium compounds.

Missiquoi Spring, Vermont, called in some literature Missisquoi Spring, is in a stright line with Clarendon Springs, Vermont, Sand Springs, Massachusetts, and Lebanon Springs, New York. The percentage of silica calculated to total solids is slightly above 10% or below 10%, since the silica of New

England silicated springs ranges from 11 to 35% of total solids.

Missiquoi Spring, Vermont, 0.0320% t. s. 6.20% $SiO_2$ and 14.16% Ca.

Clarendon Spring, Vermont,, 0.0374% t. s. 3.75% $SiO_2$ and 13.83% Ca.

Sand Springs, Massachusetts, tepid 75°F. 0.0195% t. s. 11.12% $SiO_2$ and 12.39% Ca.

Lebanon Springs, New York, tepid 75°F. 0.0413% t. s. 13% $SiO_2$ and 6% Ca.

Missiquoi Spring is mentioned by James K. Crook with Sheldon Springs, Franklin County, Vermont. There are claimed four springs:

Central Spring within the village;

Vermont Spring half a mile from the village;

Missiquoi Spring one mile and a half northward;

Sheldon Spring two miles from the village. Crook claims the older analysis advertised for the Sheldon Spring.

The northwestern corner of Vermont contains Ordovician rocks. This a balneological field of the neighborhood of Lake Champlain. The geological conditions there have caused the mineral springs to contain a large amount of calcium sulfate, also reduction products, such as sulphides and free hydrogen sulphide. We will find this the general condition of northern New York and overlaps the questioned small northwestern part of New England, in the neighborhood of Lake Champlain. To this kind of springs belongs, within Vermont: Alburgh Spring, Highgate Spring and Newburg Spring.

Alburgh Springs, Grand Isle County, Vermont, 0.0652% t. s. free sulphurhydrogen..

Highgate Spring, Franklin County, Vermont, with two contradictory analyses.

Newbury Spring, Orange County, Vermont, without analysis.

The centre of the Adirondacks and the Blue Ridge of the Appalachian system form an uncontinuous ribbon of precambric intrusive rocks. But the Adirondacks do not contain any claimed mineral spring.

### AREA IV. District 2.

### THE THERMAL AND THE CARBONATED WATERS OF NORTHEAST.

The western seashore of the Appalachian island of precambric eras became a broad fault for rising volcanic material. This fault is still in existence and duplicates in different parallel faults, all of diagonal direction on the globe and with a tendency to S-shape configurations.

This old precambric volcanic fault is the most important district for the magmatic balneology of the eastern United States. It is an almost straight line from Cape Farewell, Greenland, to Carrollton, Georgia. The northern part from Greenland to the Adirondacks was a chain of volcanic spots on a southeastern seashore of an old continent. It crosses the southern part of the State of New York without surface evidence at the present time, and in the Appalachian Mountains becomes a continuous ribbon of intrusions with two bifurcations. There in palaeozoic era it formed the northwestern seashore of a peninsula.

For this reason we will find in the third district the same geological deposits west of the southern part in question, as we found in the previous district in an eastern direction.

The precambric bifurcations mentioned are included in the first area as a field near Philadelphia, and in the third district of second area as a larger balneological field of Virginia and North Carolina.

In the fourth district of first area and third district of the second area, we learned the younger jurassic intrusions. Their efficiency concerning temperature was exhausted long ago, and does not produce any thermal or carbonised waters. The balneological field of Charleston, South Carolina, may be an exception. Within the line of the precambric intrusions we still find thermal waters in an eastern fault, and carbonised waters in a western fault. This divides the district in the thermal Tahonic field and the carbonised Saratoga field.

We may call the districts of precambric intrusions of eastern United States the districts of the Blue Ridge. In locations with very numerous mineral springs, we usually find a more central field of springs with increased temperature. In young volcanic districts this temperature is generally very high. We will learn this repeatedly in the seventh area. In old volcanic districts it is not so high. It is astonishing that thermal waters of the east are in connection with the oldest intrusions. The thermal springs centre belonging to the system of Adirondack Mountains is situated near Mount Berlin. Sand Springs, Massachusetts, and Lebanon Springs, New York, and maybe others not yet described, are in a district known as Taconic Mountains with a central point in Mount Berlin.

Sand Springs, Massachusetts, is a tepid spring of 24°C. and Lebanon Springs, New York, of 23°C. Sand Springs contain 0.0195% t. s. with 11.12% $Si0_2$ and 12.39% Ca., and Lebanon Springs 0.0413% t. s. with 13% Silica and 6% Calcium. Sand Spring is a submineralised tepid spring, and Lebanon Spring is a tepid low average mineralised spring.

The Tahonic Mountains are situated in the northern continuation of the Blue Ridge, but there is no evidence of volcanic intrusion and especially no evidence of precambric intrusion.

Waubeeka Spring at South Williamstown, Massachusetts, is a cold spring of the Tahonic Mountains with 0.0055% total solids, 67% earthy salts with predominant calcium carbonate. Silicon is only 6% of total solids and excludes this spring from the low mineralised New England springs which range from 11 to 35% silicon. It is also an exception from the springs of the following district of the fourth area overleaping to New England and containing 0.02 to 0.07% total solids. If Wawbeeka Spring should be separated from the tepid springs of the Tahonic Mountains, we have to unite it with the sixth district of the first area, especially with the balneological field of southeastern New York. This shows the location of Wawbeeka Spring at the triboundary point of first, third and fourth area. The magmatic waters reaching in tepid condition the neighboring surface became somewhat higher mineralised toward the northeast at Williamstown, corresponding to the character of the New England area. South of Mount Berlin, unknown tepid springs may be situated as first springs of the first area. Toward east Lebanon Springs is the first spring of the fourth area and shows the character of fourth area by the double mineralisation in comparison with Sand Springs.

The sodium chloride field of the next district leaps in the east over to the presilurian volcanic ribbon, and there is crossed by a fault producing magmatic carbonic acid gas. The mineral springs there are Sodium Chloride Carbonated springs. This is the field we hear so much about—that of Saratoga Springs and the vicinity. The original magmatic waters of the Saratoga fault without the high sodium chloride mineralisation belong to the southern district of the Blue Ridge.

In the Saratoga field there is a well studied geological fault in the direction of northeast to southwest. This fault is known to geologists as Hoffman's fault; this fault is curved and slightly S formed. It is rectangularly intersected by weaker faults. This may be seen by the many rectangular knees of creeks and rivers. The most remarkable springs of this fault are the mineral springs of Saratoga Springs and Ballston Spa conglomerated at three fault intersections. A weaker parallel fault, with less known carbonated springs is a few miles distant to the east. The relationship of these different springs to the Hoffmann's fault is explained by Cushing and Ruedemann.

At Saratoga Springs, the basis are precambric Crystalline rocks. They are covered by Potsdam Sandstone, then Theresa formation, and on the top Little Falls Dolomite with the petrographic character of Hoyt-lime poor in magnesia. The strata are slightly declined. The faults have the direction of "Bstreichen." The angle of decline is not important.

The uppermost stratum of Ordovician, called black Canajoharie shale, is the surface rock where the Saratoga fault creates mineral springs.

At Ballston Spa two diagonal faults and a rectangular fault intersect with 90° and 45° about. The middle part of direction of Kayanderosseras River shows ordinate and abscisse. A parallel ordinate is indicated by the northern knee of the creek running to Loughberry Lake.

To the Hoffman's fault also belong Gurn Spring situated to the north. Between Gurn Springs and Saratoga Springs the other fault intersection mentioned must be suggested. This point is still undeveloped.

Quaker Springs belong to the weaker fault. The missing links of the neighboring springs are Adirondack Mineral Spring near Whitehall, and Reid's Mineral Spring near South Argyle, both in Washington County, New York. Albany Artesian Well with 1.05% total solids in Albany County also belongs to the Saratoga group. Franklin Springs with 0.6% total solids leads to the Syracuse field of the next district.

There is mentioned south of Saratoga Lake: White Sulphur Spring with a bathing pavilion.

The Hoffmann's fault is parallel to the Appalachian elevation and gets its principal northern concave curvation by the central point of the Adirondacks intrusion.

AREA IV District 3.

### THE NORTHEASTERN SILURIAN AND DEVONIAN DEPRESSION.

The northeastern Silurian and Devonian depression of the eastern Mississippi area includes the principal part of the State of New York, except the mountain region of the Adirondacks, and the southeastern part of the state, but including the southeastern part of Ontario and the middle northern part of Pennsylvania.

It is the principal district of distribution of the Buffalo Medical Journal. It is very important for the American balneology and creates the special interest of the readers of our journal toward balneology.

The depression between the Appalachian Mountains, Ozark Mountains and Wisconsin usually shows palaeozoic deposits. The strata there do not occur thoroughly concordant. The area became transitorialy and locally dry in oldest palaeozoic era forming rock salt deposits. Later palaeozoic sections of a second submersion contain a high amount of calcium compounds and other earths. These sodium compounds and calcium compounds are more or less soluble. The state of New York and neighboring parts show this condition in the district questioned. The same conditions repeat southwest of the Wisconsin district in a relatively small ribbon.

Most of the mineral waters concerned are located in the Albany-Buffalo line. We learned that the waters of New England were of an overoxydised character, where they contain juvenile waters. and of a silicic character, where the origin of mineralisation is situated on the surface. We have to expect ascending magmatic waters in the Albany-Buffalo line. Such waters of oxydising character passing different strata which contain sulphur compounds and earthy carbonates lead to very complicated chemical reactions. Sulphur compounds may be oxydised to sulphuric acid and its compounds. A resulting excess of sulphuric acid may lead to deliberation of carbonic acid gas. A mixture containing carbonic acid gas, residues of organic substances, for instance crenic acid and its compounds, and sulfuric acid, may lead to a reduction forming hydrogen sulphid. The presence of iron may lead to formation of iron sulfates, to secondary precipitation of iron oxydul oxyde and may form acid sulfate springs. All these kinds of mineral springs are found in the upper state of New York.

The sodium chloride springs correspond to the older deposits. The real variation reaches a higher degree in younger palaeozoic sections. There we have to consider Ordovician to Devonian.

Chemically deliberated or maybe magmatic carbonic acid impregnating a sodium chloride extract of high concentration, we had already found in the Saratoga field. Farther to the east, we had already found reduced sulfide springs of the small field of Lake Champlain.

Therein occur two distinct types of mineral springs. They contain either more than 1% sodium chloride or 0.1 to 0.2% calcium sulfate calculated to water or an intermediary mineralisation or secondary derivatives.

For this reason the third district has to be subdivided into the field of brines, the different fields of sulfide waters, and the field of iron and acid iron waters.

### The Brine Field of the Third District.

Between Albany and Buffalo, but north of this line, and with the center in Syracuse, New York, are large rock deposits of sodium chloride of old geological origin. In this field they have some mineral springs belonging to the group of common sodium chloride springs. A southern offspring is the different springs of Glen Springs, New York.

St. Catharine's Well is a Canadian Sodium chloride water.

Different Sulfide Fields.

From Albany to Buffalo, the depression of the transcontinental fault enforced different crossing rivers to follow this line for a certain length. In the palaeozoic era and later there were many long narrow lakes connected by rivers with falls similar to the Niagara River, but lakes and rivers in a continuous straight line. Later the lakes on the plains of the Albany-Buffalo line became filled with detritus and drift. The Oneida Lake is the only concerned lake persisting. It was an easy pre-existing line for the construction of railroads and the excavation of the canal. The geological condition there as well, favors an accumulation of valuable mineral springs where the original rocks reach the surface, for the increased amount of inhabitants and travelers offered a better chance to find such springs.

In the Albany-Buffalo line, south of the 43° latitude is a large number of mineral springs, with about 0.15 to 0.35% total solids. More than half of these solids are ordinarily calcium sulphate. The amount of free and compound hydrogen sulfide is low, mostly below 0.01% weight.

West of Utica the sulfur springs are really within 43° latitude. East of Utica the boundary of Devonian and Silurian to the east bends east-south-east, and south-west from Albany bends again to a straight southerly direction. For this reason the sulfur springs of Otsego County and Schoharie County are about on the average of 20 kilometres more southward.

---

**Local Anaesthesia in Surgery of the Colon and Rectum,** Wm. M. Beach, International Clinics. 1. Eliminating terrorism associated with operations under general anaesthesia. 2. Absence of post-operative distress and complications. 3. The Anaesthesia is complete, thoroughly blocking the field, thus preventing shock. 4. It persuades the patient to undergo an operation because the detention from business is shorter and post-operative pain is less. 5. Skill in technic is achieved by virtue of the surgeon's care in gentle handling of a conscious patient. 6. It will teach him to handle tissues more deftly in general anaesthesia realizing that much pain and tendency to infection follows tearing and mutilating of soft parts. 7. Local anaesthesia conserves the patient's peace of mind, as there are many who will testify to its efficiency and complete relief with so little inconvenience.

